# Supplementary material for: Diagonal nematicity in the pseudogap phase of HgBa2CuO4+δ
Source: Nat Commun. 2019 Jul 23;10:3282. doi: 10.1038/s41467-019-11200-1 (PMC6650423; doi:10.1038/s41467-019-11200-1)
Supplement: Supplementary file 1 — Supplementary Information [file 41467_2019_11200_MOESM1_ESM.pdf]

Supplementary Information: Diagonal Nematicity in the Pseudogap Phase of $HgBa_2CuO_{4+\delta}$

H. Murayama[1], Y. Sato[1], R. Kurihara[1], S. Kasahara[1], Y. Mizukami[2], Y. Kasahara[1], H. Uchiyama[3,4], A. Yamamoto[5], E.-G. Moon[6], J. Cai[7], J. Freyermuth[7], M. Greven[7], T. Shibauchi[2], Y. Matsuda[1]

[1] *Department of Physics, Kyoto University, Kyoto 606-8502 Japan*

[2] *Department of Advanced Materials Science, University of Tokyo, Chiba 277-8561, Japan*

[3] *Materials Dynamics Laboratory, RIKEN SPring-8 Center, 1-1-1 Kouto, Sayo, Hyogo 679-5148, Japan*

[4] *Research and Utilization Division, Japan Synchrotron Radiation Research Institute (SPring-8/JASRI), 1-1-1 Kouto, Sayo, Hyogo 679-5198, Japan*

[5] *Graduate School of Engineering and Science, Shibaura Institute of Technology, 3-7-5 Toyosu, Koto-ku, Tokyo, 135-8584, Japan*

[6] *Department of Physics, Korea Advanced Institute of Science and Technology, Daejeon 305-701, Korea*

[7] *School of Physics and Astronomy, University of Minnesota, Minneapolis, Minnesota 55455, USA*

This file includes:

Supplementary Figure 1

Supplementary Figure 2

Supplementary Figure 3

Supplementary Figure 4

Supplementary Figure 5

Supplementary Figure 6

Supplementary Figure 7

Supplementary Figure 8

Supplementary Figure 9

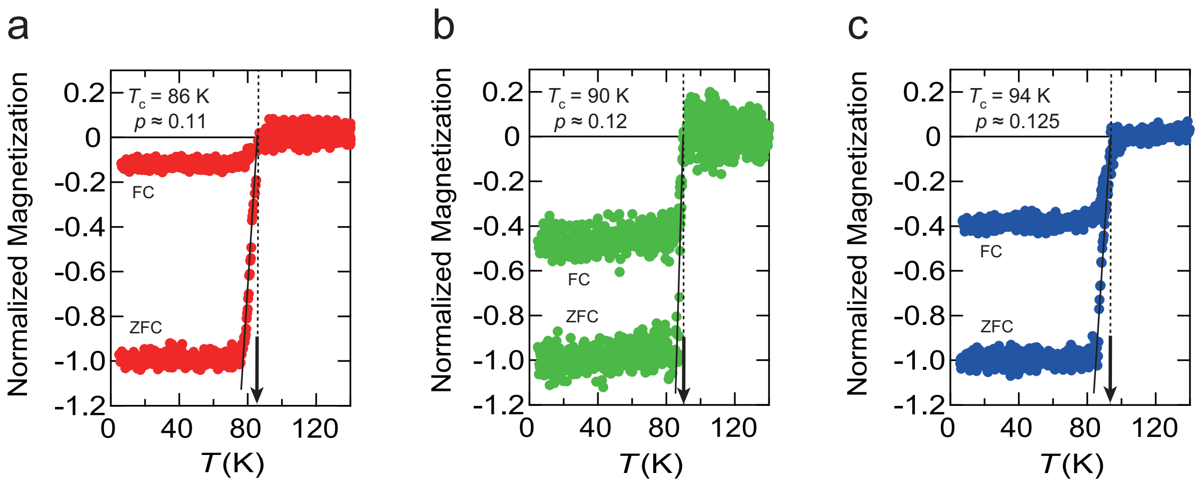

Supplementary Figure 1 | Superconducting transition in Hg1201 for different doping levels. **a**, **b**, **c**, Temperature dependence of normalized magnetization $M(T)/|M(5\,\mathrm{K})|$ for $p \approx 0.11$ (**a**), 0.12 (**b**) and 0.125 (**c**), respectively. The magnetization is measured under field-cooling (FC) and zero-field-cooling (ZFC) conditions in a magnetic field of 10 Oe applied along the c-axis. Arrows indicate T_c determined by the onset of diamagnetic signal.

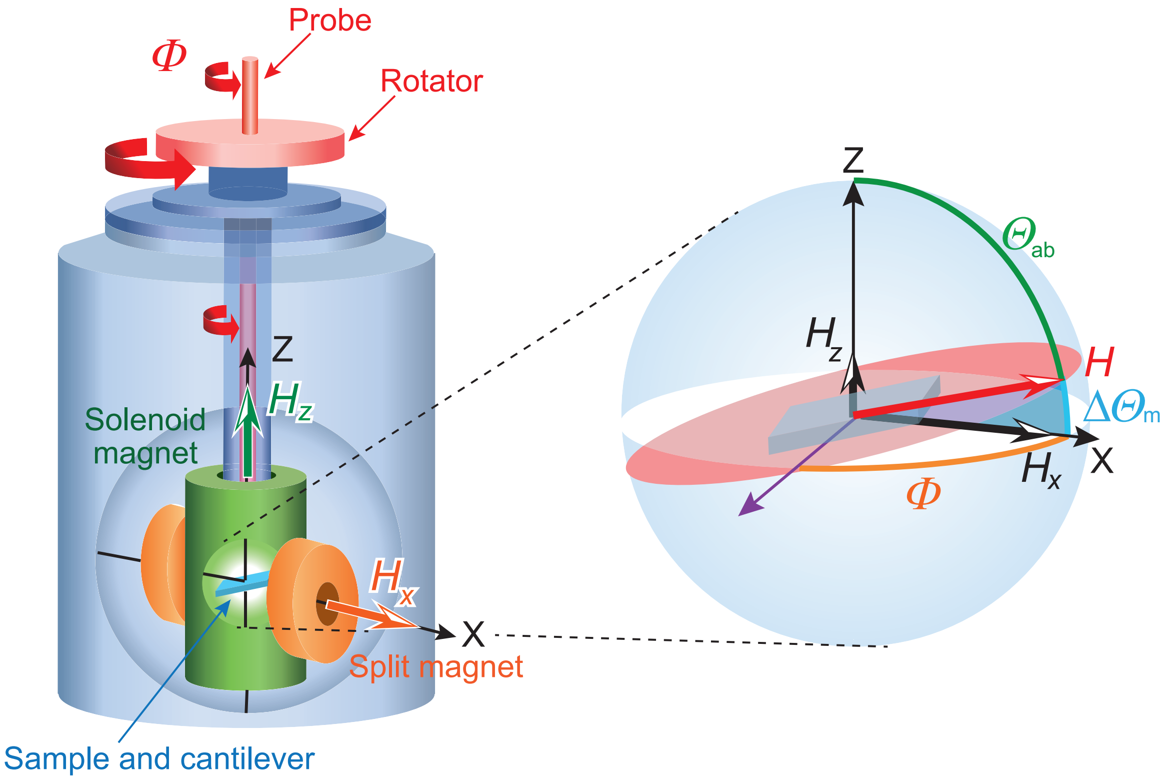

Supplementary Figure 2 | Schematic figures of the torque measurement system. Left: Schematic of the experimental apparatus. To apply the magnetic field **H** relative to the crystal axes with high accuracy, we used a system with two superconducting magnets to generate field components, H_X (orange arrow) and H_Z (green arrow), in two mutually orthogonal directions (X and Z), and a mechanical rotation stage at the top of the dewar. The whole sample probe, which is in a variable temperature insert, is rotated around the Z axis as indicated by the red arrows. Right: Schematic of the in-plane Φ-scan measurements. Θ_{ab} is the angle between the Z axis and the crystal ab plane. $\Delta\Theta_m$ is the misalignment away from H_X, which is eliminated at each Φ by 2D vector field. (see Supplementary Fig. 3).

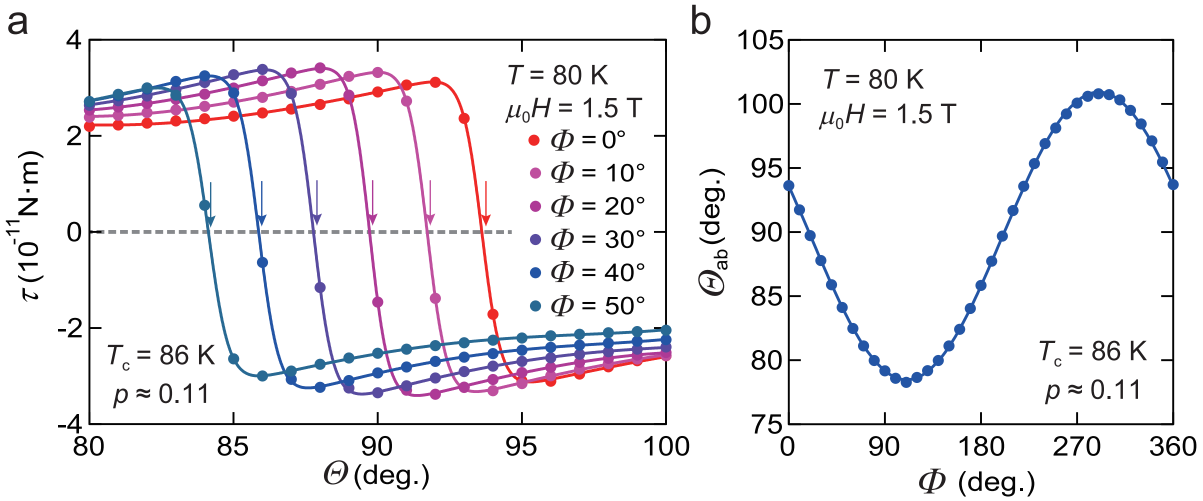

Supplementary Figure 3 | Determination of the sample alignment. **a**, Torque τ plotted as a function of angle Θ from the Z axis for $p \approx 0.11$ in the superconducting state at $T = 80$ K in a magnetic field of $\mu_0 H = 1.5$ T rotated across the ab plane at several Φ. The torque curve is completely reversible as a function of Θ at this temperature. τ abruptly changes sign when crossing the ab plane. The arrows indicate the directions of the ab plane, Θ_{ab}, at which τ vanishes. **b**, Θ_{ab} plotted as a function of Φ. Θ_{ab} is nearly perfectly sinusoidal as a function of Φ. To precisely rotate $\mathbf{H}$ within the ab plane, the misalignment of the ab plane w.r.t. the XY plane, $\Delta\Theta_m = \Theta_{ab} - 90°$, is eliminated at each Φ via computer control of the vector magnet and mechanical rotator. Subsequently, the in-plane torque $\tau(\phi)$ is measured with a field misalignment smaller than 0.1 deg.

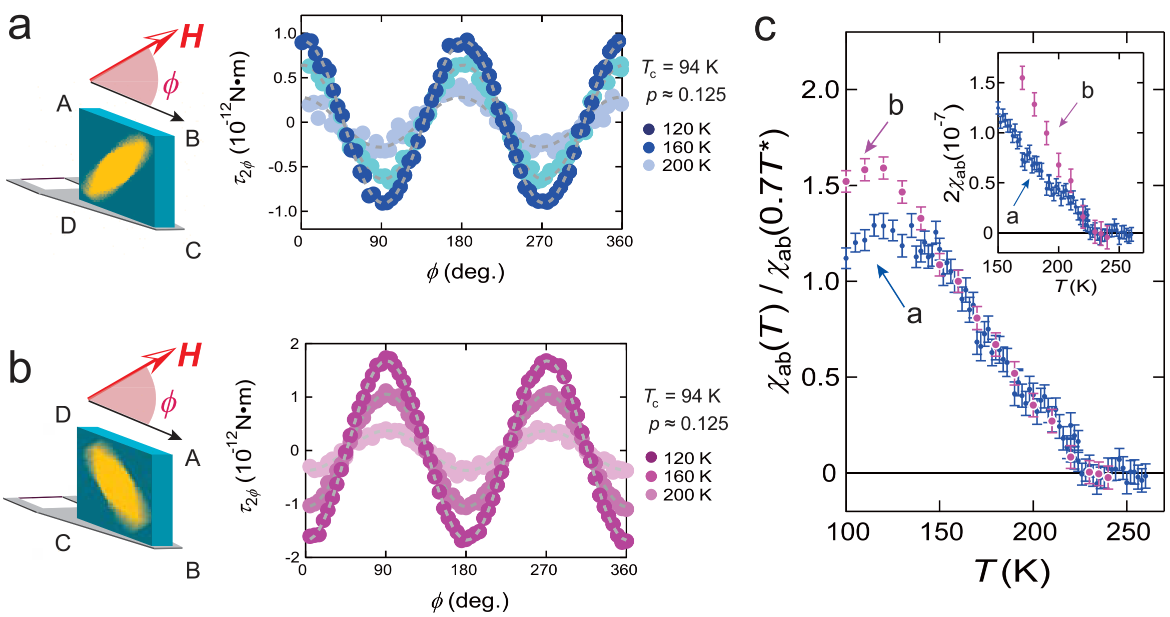

Supplementary Figure 4 | The influence of strain on the side of the crystal attached to the cantilever. a, **b**, Left: Schematic of torque measurements in two different configurations. Yellow ellipses indicate the nematic directions. In **b**, the torque is measured after remounting the crystal rotated by 90 deg relative to the configuration illustrated in **a**. Right figures depict the respective $\tau_{2\phi}$ vs. ϕ data for the crystal with $p \approx 0.125$. The direction of the nematicity is unchanged relative to the crystal axes after the crystal rotation. **c**, Inset depicts temperature dependence of $2\chi_{ab}$ for configurations shown in **a** and **b**. Main panel depicts $\chi_{ab}(T)/\chi_{ab}(0.7T^*)$ for both configurations. Error bar represents s.d. of the sinusoidal fit to the $\tau_{2\phi}(\phi)$ curves. The temperature dependence is essentially the same down to 150 K below which short-range CDW order appears.

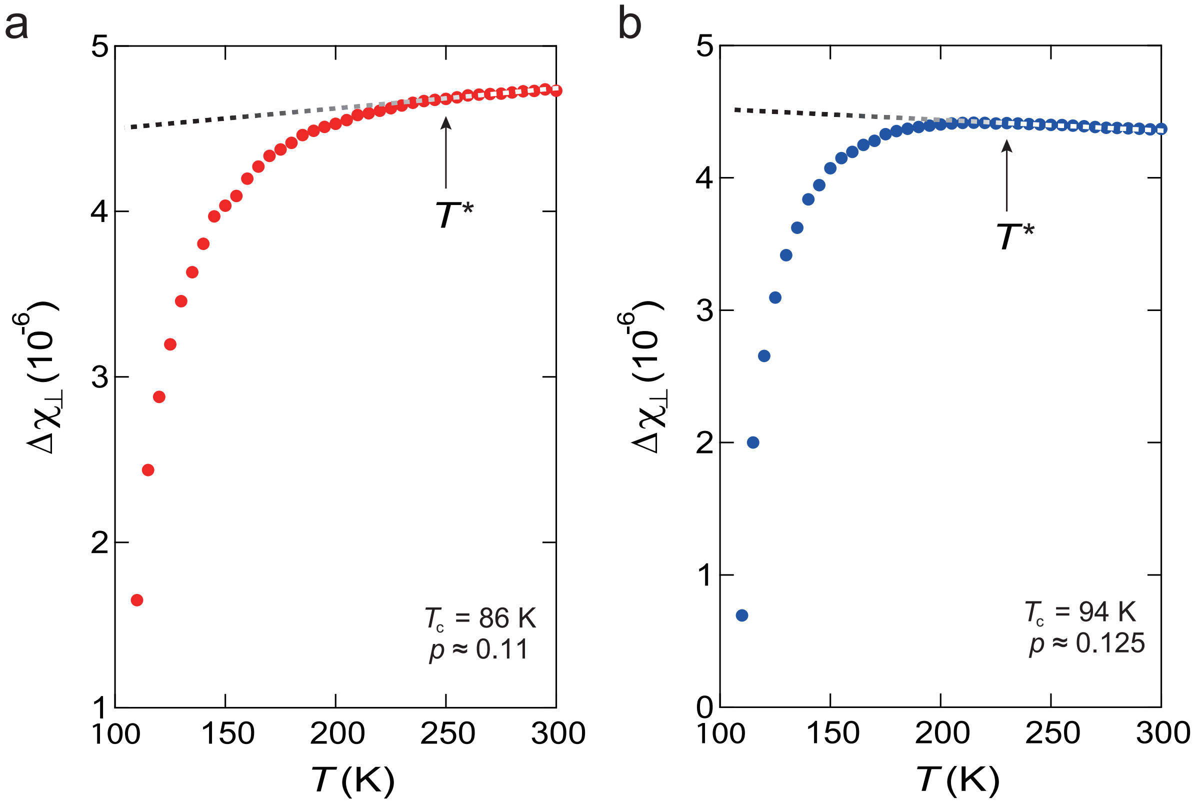

Supplementary Figure 5 | Out-of-plane anisotropy of the magnetic susceptibility in Hg1201. Temperature dependence of the out-of-plane anisotropy of the magnetic susceptibility, $\Delta\chi_\perp \equiv \chi_{cc} - \chi_{aa}$, determined from the $\tau(\theta)$ curves for $p \approx 0.11$ (**a**) and 0.125 (**b**). Dashed lines are T-linear fits at high temperatures. Below T^*, $\Delta\chi_\perp$ deviates from the high-temperature behaviour.

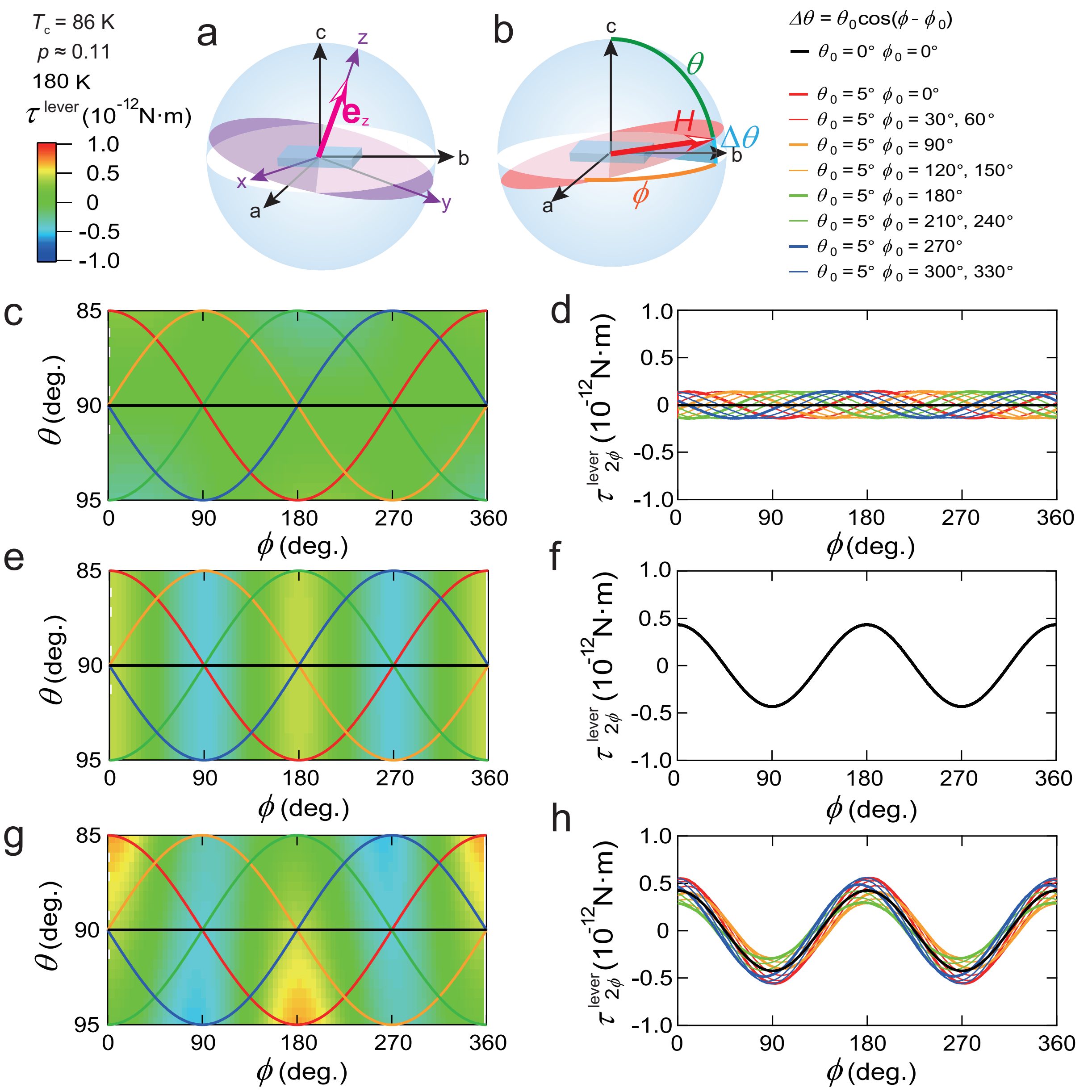

Supplementary Figure 6 | Expected magnetic torque for the cases with/without in-plane anisotropy. a, Schematic of mount misalignment between sample and cantilever. The crystal axes are labeled by a, b, and c. Alignment of the cantilever is indicated by xyz coordinates, where the cantilever probes torque along the z axis for the bending within the xy plane (shown in purple). The normal unit vector of the bending plane is indicated by $\mathbf{e}_z$. **b**, Schematic of alignment between sample and field rotation plane (shown in red). The misalignment of the magnetic field w.r.t. the ab plane is given by $\Delta\theta = \theta_0 \cos(\phi - \phi_0)$. **c**, Expected torque amplitude, $\tau^{\text{lever}} = \tau \cdot \mathbf{e}_z$ as a function of field misalignment (θ, ϕ) calculated for case (A), as discussed in Methods. The calculations are performed for $p \approx 0.11$ using the

out-of-plane anisotropy, $\chi_{cc} - \chi_{aa}$, at 180 K. Mount misalignment between the sample and the lever is included. Diagonal nematicity χ_{ab} is supposed to be absent in this calculation. Colours represent the expected torque amplitude τ^{lever}. **d**, Expected angular dependence of the torque, $\tau_{2\phi}^{\text{lever}}$, for the trajectories in **c** with various field misalignments. **e**, **f**, Same plots for $p \approx 0.11$ at 180 K for case (B) (see Methods). In this calculation, both out-of-plane anisotropy, $\chi_{cc} - \chi_{aa}$, and in-plane anisotropy, χ_{ab}, are included. The mount misalignment between the sample and the lever is supposed to be zero. **g**, **h**, Same plots for $p \approx 0.11$ at 180 K for case (C) (see Methods). In addition to $\chi_{cc} - \chi_{aa}$ and χ_{ab}, mount misalignment between the sample and the lever is included.

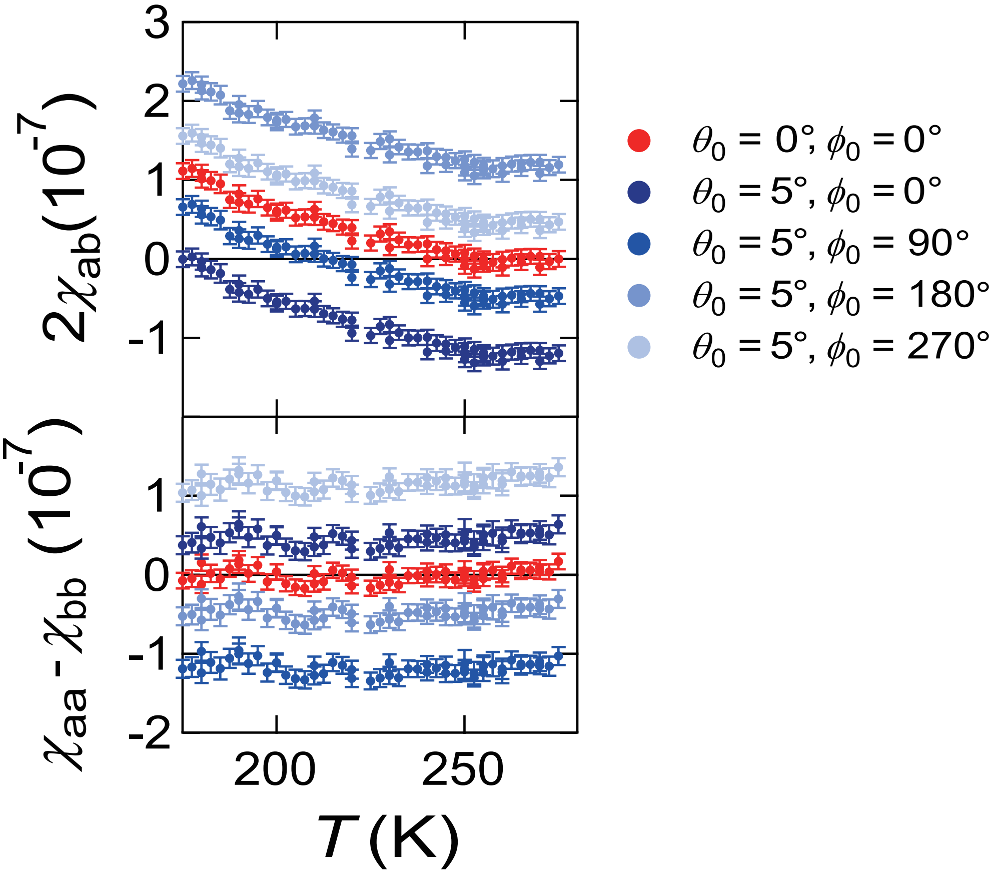

Supplementary Figure 7 | Expected temperature dependence of magnetic susceptibility anisotropies for $p \approx 0.11$ for a magnetic field rotated in a misaligned plane. Even for a large field misalignment of $\theta_0 = 5$ deg, the contribution from the out-of-plane component only appears as a slight shift of the original signal, whereas the onset of χ_{ab} at T^* is observed clearly.

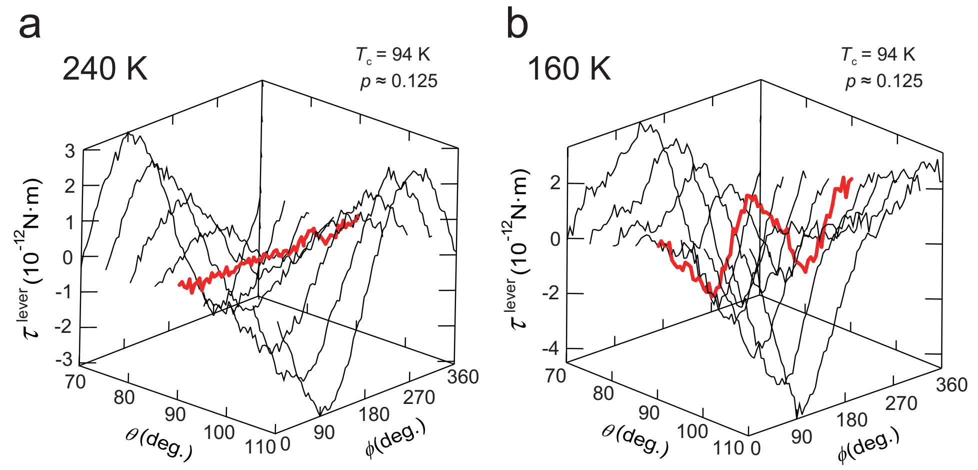

Supplementary Figure 8 | Magnetic torque for $p \approx 0.125$ under conical field rotations. Magnetic torque recorded for conical field rotations at several θ at (**a**) $T = 240$ K and (**b**) 160 K, respectively. The sample is mounted on the lever with an apparent mount misalignment of $\Theta_0 = 10.6$ deg and $\Phi_0 = 274$ deg. The emergence of the in-plane anisotropy below T^* is clearly seen at $\theta = 90\,$deg at $T = 160$ K.

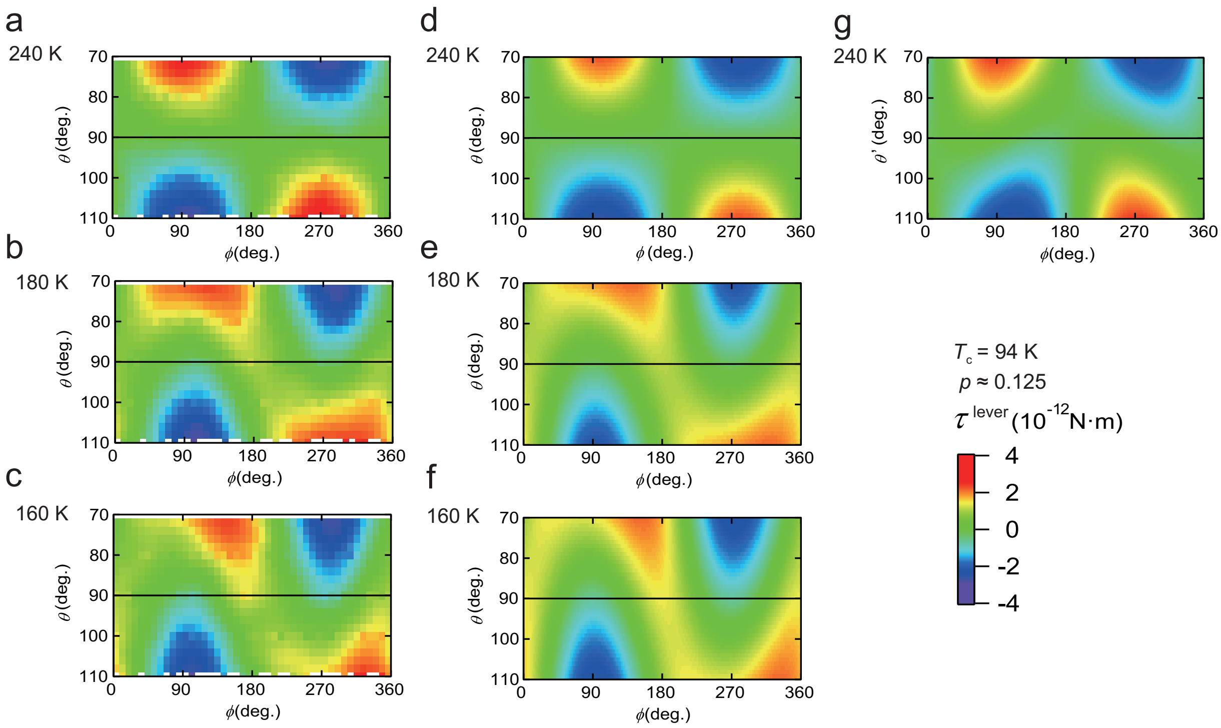

Supplementary Figure 9 | Comparisons of the observed and the expected torque under finite out-of-plane field component. **a**, **b**, **c**, The observed magnetic torque amplitude mapped in the (θ, ϕ) plane at T = 240, 180, and 160 K, respectively. Colours represent the amplitude of the magnetic torque. **d**, **e**, **f**, The expected torque response at T = 240, 180, and 160 K, respectively, when both field and mount misalignments are present. **g**, The expected torque response when the direction of the sample plane is misidentified. Here, an inclined plane of $\theta' = (\theta - 5^\circ)\cos(\phi - 20^\circ)$ is assumed as a misidentified direction of the sample plane. At T = 240 K ($> T^*$), in-plane anisotropy is absent and the torque amplitude shows symmetric behaviour in the (θ, ϕ) plane, indicating that $\theta = 90$ deg correctly captures the direction of the *ab* plane. As the in-plane anisotropy emerges below T^*, torque response becomes asymmetric in the (θ, ϕ) plane due to the mixing of two-fold oscillations of the diagonal nematicity.
